# Patient-specific vascular models for optimal catheter selection: Two aneurysm embolization cases

**DOI:** 10.1016/j.radcr.2025.02.080

**Published:** 2025-03-18

**Authors:** Ryo Morita, Daisuke Abo, Takaaki Fujii, Naoya Kinota, Daisuke Kato, Kouji Yamasaki, Motoma Kanaya, Taisuke Harada, Osamu Sugita, Norio Kawamura, Akinobu Taketomi, Kohsuke Kudo

**Affiliations:** a Department of Diagnostic and Interventional Radiology, Hokkaido University Hospital, N-14, W-5, Kita-ku, Sapporo, Hokkaido 060-8648, Japan; b Department of Dental Radiology, Hokkaido University Hospital, N-14, W-5, Kita-ku, Sapporo, Hokkaido 060-8648, Japan; c Department of Diagnostic Imaging, Faculty of Medicine, Hokkaido University, N-15, W-7, Kita-ku, Sapporo, Hokkaido 060-8638, Japan; d Institute of Health Science Innovation for Medical Care, Clinical Development Division, Hokkaido University Hospital, N-14, W-5, Kita-ku, Sapporo, Hokkaido 060-8648, Japan; e Department of Gastroenterological Surgery I, Graduate School of Medicine, Hokkaido University, N-14, W-5, Kita-ku, Sapporo, Hokkaido 060-8648, Japan; f Global Center for Biomedical Science and Engineering, Faculty of Medicine, Hokkaido University, N-14, W-5, Kita-ku, Sapporo, Hokkaido 060-8648, Japan

**Keywords:** Preoperative simulation, Patient-specific vascular model, Three-dimensional printer, Aneurysm embolization, Optimal catheter selection

## Abstract

Patient-specific vascular models enhance preoperative planning in interventional radiology, particularly in complex and anatomically challenging cases. This report presents two cases of complex aneurysm coil embolization in which three-dimensional, printed, patient-specific vascular hollow models were key to selecting optimal catheters, leading to successful interventions. Preoperative simulations with these models facilitated preselection of the optimal catheter, minimizing the need for intraoperative catheter exchange and reducing the time required for catheter engagement and placement. This approach improves procedural efficiency and outcomes by ensuring a smoother workflow.

## Introduction

Coil embolization of peripheral aneurysms in the trunk region has been widely practiced [1]. Achieving successful outcome requires sufficient catheter stability and the precise placement of an appropriate catheter in the primary branch of the aorta. However, preoperative computed tomography (CT) imaging often fails to provide adequate information to determine whether the chosen catheter shape will be compatible with the primary branch originating from the aorta, such as the celiac artery, superior mesenteric artery (SMA), inferior mesenteric artery, or bronchial artery [2]. Consequently, multiple catheter exchanges during the procedure may become necessary, leading to prolonged procedure times and increased risk of vascular intimal injury, particularly when using large-diameter guiding catheters [3].

Preoperative simulation using patient-specific vascular models offer a potential solution by reducing the need for catheter replacement, shortening the time required for catheter engagement and placement, and minimizing intimal damage risk. In Interventional Radiology, several reports have discussed preoperative simulations of aneurysm embolization using individualized vascular models [[4], [5], [6]]. However, most existing models either lack hollow structures and only replicate anatomical shapes [4] or are small-scale hollow models that replicate only aneurysms and adjacent inflow and outflow vessels [[5], [6], [7]]. These models fail to reproduce the extended access route from the femoral, radial, or brachial arteries. Furthermore, hollow models often lack flexibility [5,8] and differ in physical properties, such as hardness and slipperiness from biological arteries. Consequently, these models are insufficient for simulating catheter insertion into the primary branches of the aorta.

To address these limitations, we developed a transparent, flexible, and highly slippery vascular model that accurately reproduced a wide range of vascular structures [9]. Its utility has been demonstrated in procedures involving renal arteriovenous malformations [10]. Preoperative simulations using this model have been reported to improve the embolization material selection and device access, including identifying the most suitable catheter and guidewire [10]. Therefore, we planned to use preoperative simulation using this model to treat peripheral aneurysms and aimed to confirm the optimal catheter shape preoperatively.

Herein, we report 2 anatomically complex cases of peripheral aneurysms. In both cases, preoperative simulations using the developed model were performed to select the optimal catheter shape for successful treatment.

## Case presentation

### Preoperative simulation using patient-specific hollow vascular models

Our institutional review board approved the use of vascular models for this retrospective case report. Written informed consent was obtained from the patient for publication of this case report and any accompanying images. Consent for publication was obtained for every person's data included in the study. Digital Imaging and Communications in Medicine (DICOM) data from three-dimensional (3D) computed tomographic angiography (CTA) images, with a slice thickness of 0.5 mm, were converted to stereolithography (STL) files. The hollow feature of the model was created using MeshMixer (Autodesk, San Rafael, CA) with a wall thickness of 1.0 mm. The data were processed using PreForm software (Formlabs, Somerville, MA), and the model was printed using a Form 3L 3D printer with flexible, transparent materials (Flexible 80A; Formlabs). The models were subsequently rinsed with isopropyl alcohol, dried, and cured [9,10].The vascular models were coated with silicone and connected to a sheath in a white box to simulate endovascular procedures without fluoroscopy [9].

### Case 1

**Fig. 1:**
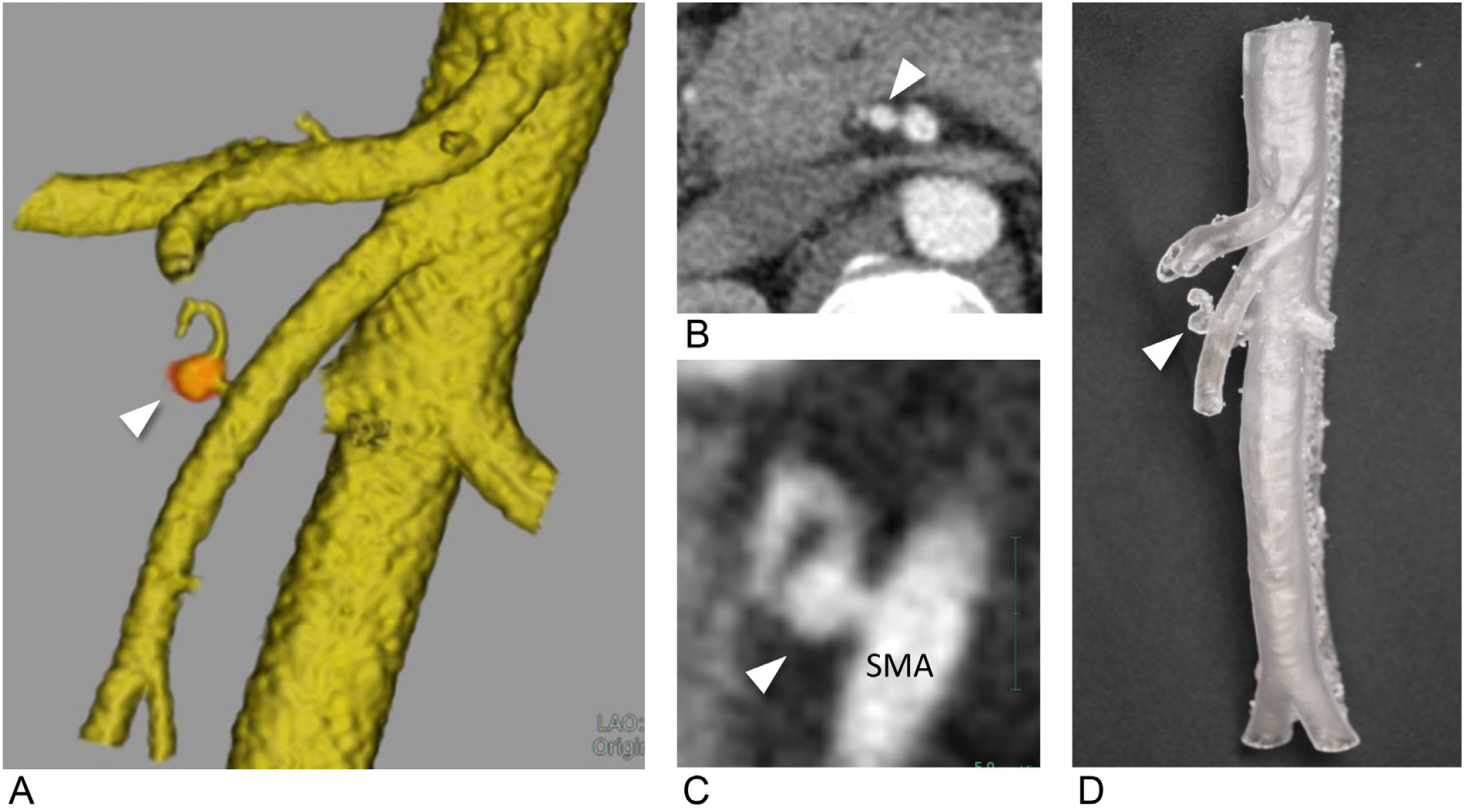
A 40-year-old female with a dorsal pancreatic artery aneurysm (Case 1). A 5 × 5 mm saccular aneurysm (white arrowhead) at the dorsal pancreatic artery originated from the superior mesenteric artery (SMA). Performing the filling coils without migrating from the SMA was challenging. (A) Volume rendering of computed tomography (CT) angiography showing a 5 × 5 mm saccular aneurysm (white arrowhead), (B) (C) CT angiography showing a 5 × 5 mm saccular aneurysm (white arrowhead), short neck to the SMA. (D) Transparent and flexible patient-specific hollow vascular models with dorsal pancreatic artery aneurysm (white arrowhead).

A female patient in her 40s presented with a 5 × 5 mm saccular aneurysm (Fig. 1A-C) at the origin of the dorsal pancreatic artery, branching from the SMA. She was referred to our department for prevention of aneurysm rupture. The proximity of the aneurysm to the SMA trunk made it challenging to perform coil embolization without risking coil deviation into the SMA trunk, particularly during the coil-finishing phase. Given these anatomical complexities, we created a patient-specific vascular model (Fig. 1D) to simulate the procedure and test the various catheter options.

**Fig. 2:**
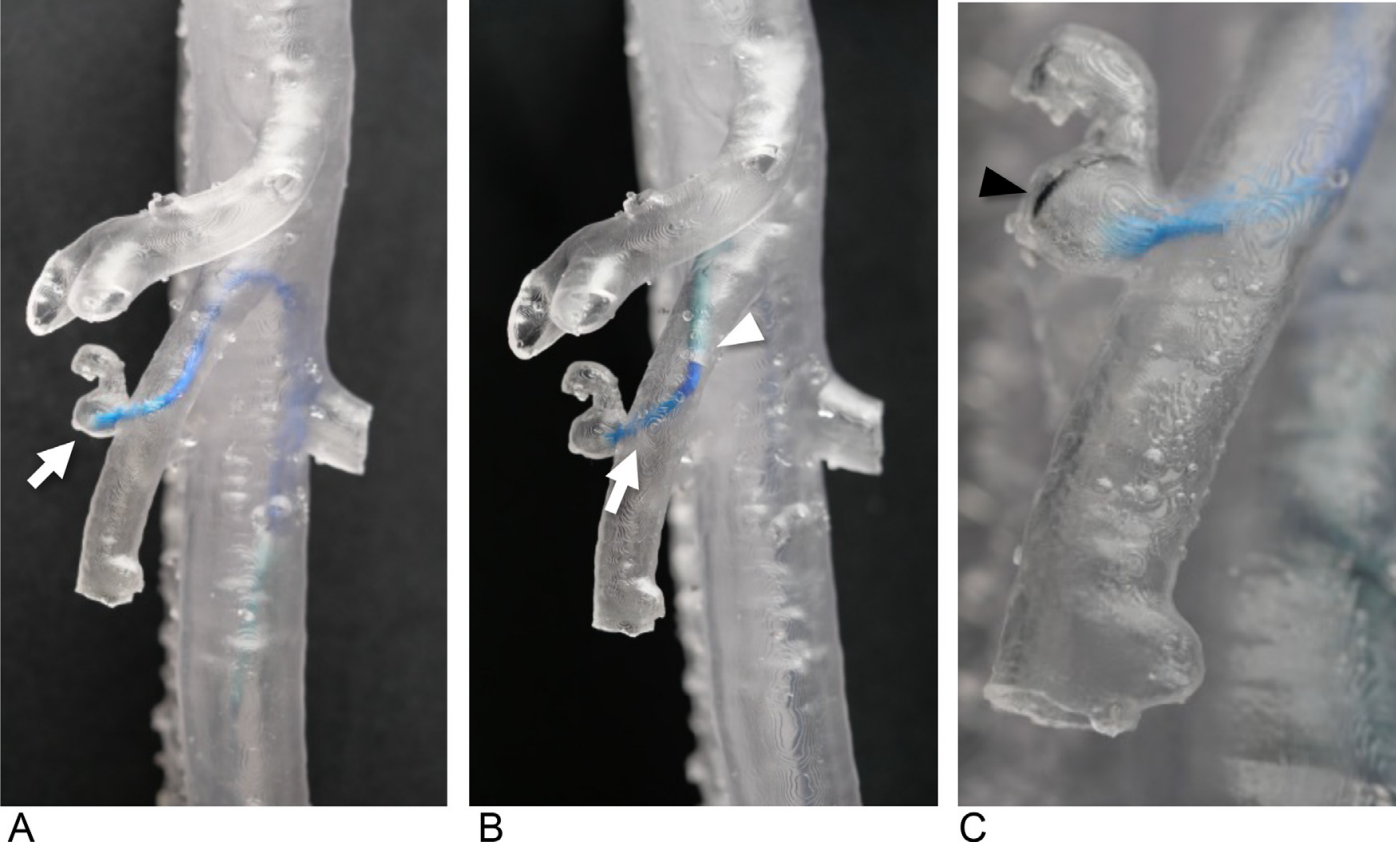
Preoperative simulation using transparent vascular models without fluoroscopy (Case 1). (A) The 4-Fr Rösch hepatic (RH) catheter (white arrow) advanced to the saccular aneurysm. (B) The 4-Fr RH catheter (white arrow) with a 4.5-Fr shepherd hook type guiding sheath (white arrowhead). (C) A 0.016-inch wire (black arrow) is inserted into the aneurysm via a microcatheter with a 4-Fr RH catheter.

Through the simulation, it was determined that using a 4.5-Fr guiding sheath (Parent Plus45 shepherd hook [SH] type, Medikit) and a 4-Fr Rösch hepatic (RH) catheter would provide sufficient backup support to ensure accurate coil placement (Fig. 2).

**Fig. 3:**
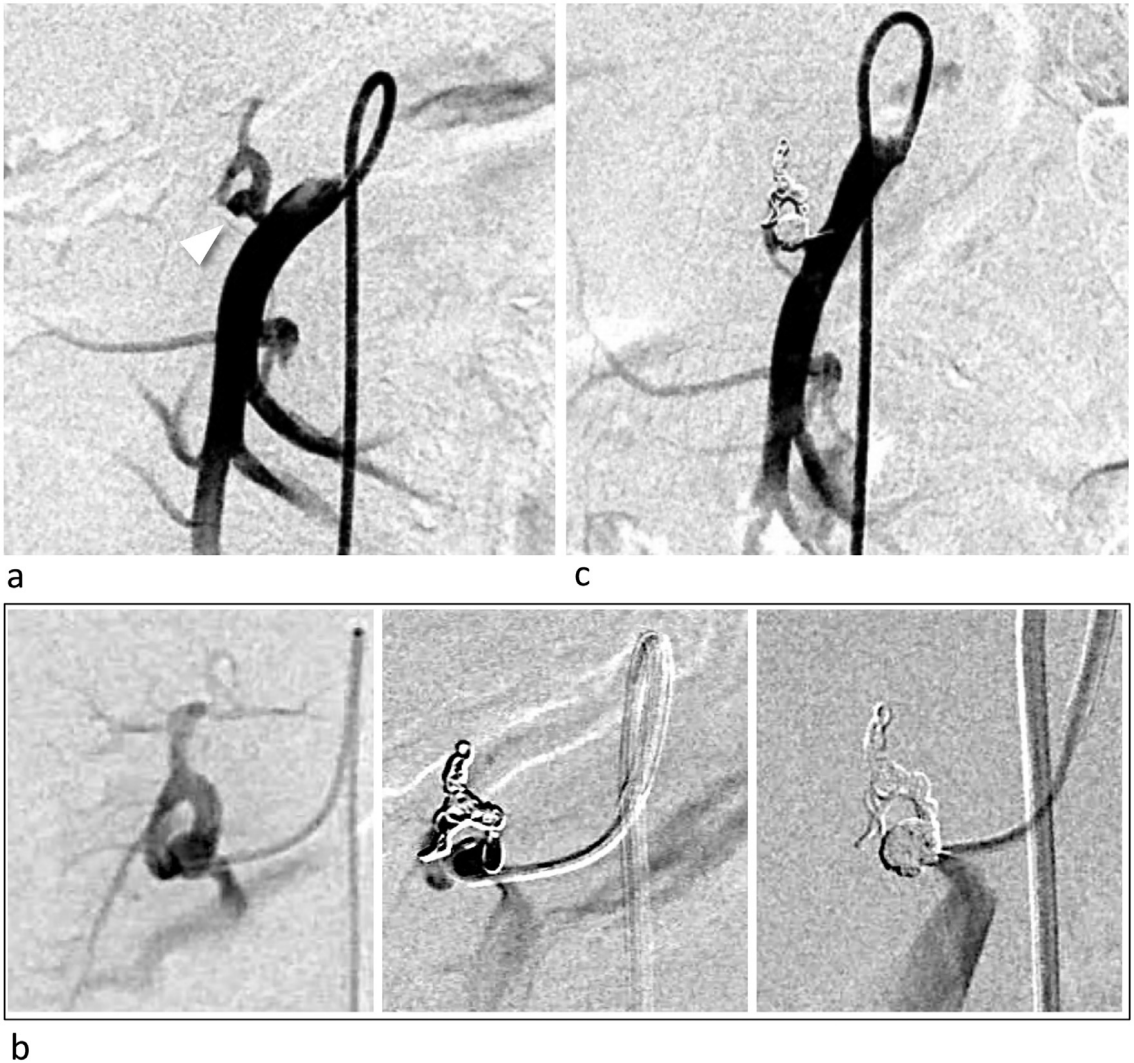
Digital subtraction angiography (DSA) during the treatment (Case 1). (A) DSA demonstrating a saccular aneurysm (white arrowhead) before treatment. (B) Coil embolization (distal embolization and packing) was performed via a microcatheter, and the right panel shows complete occlusion of the aneurysm and the shirt neck via the superior mesenteric artery (SMA). (C) DSA after treatment demonstrated complete disappearance of the aneurysm, and the SMA was patent.

During the actual procedure in the patient, the same catheters (Parent Plus45 SH type and 4-Fr RH catheter) were used via the right femoral approach. Coil embolization for distal embolization and packing was performed with 2.4/2.6-Fr Leonis Mova (125 cm, Sumitomo Bakelite, Tokyo, Japan) inserted into the distal artery to the dorsal pancreatic artery aneurysm using detachable coils (Target XL Detachable Coils, Stryker Corporation, Kalamazoo, Michigan, USA; 3 mm × 9 cm × 1, 4 mm × 12 cm × 1, 5 mm × 20 cm × 1). Embolization was successfully completed with no complications (Fig. 3).

### Case 2

**Fig. 4:**
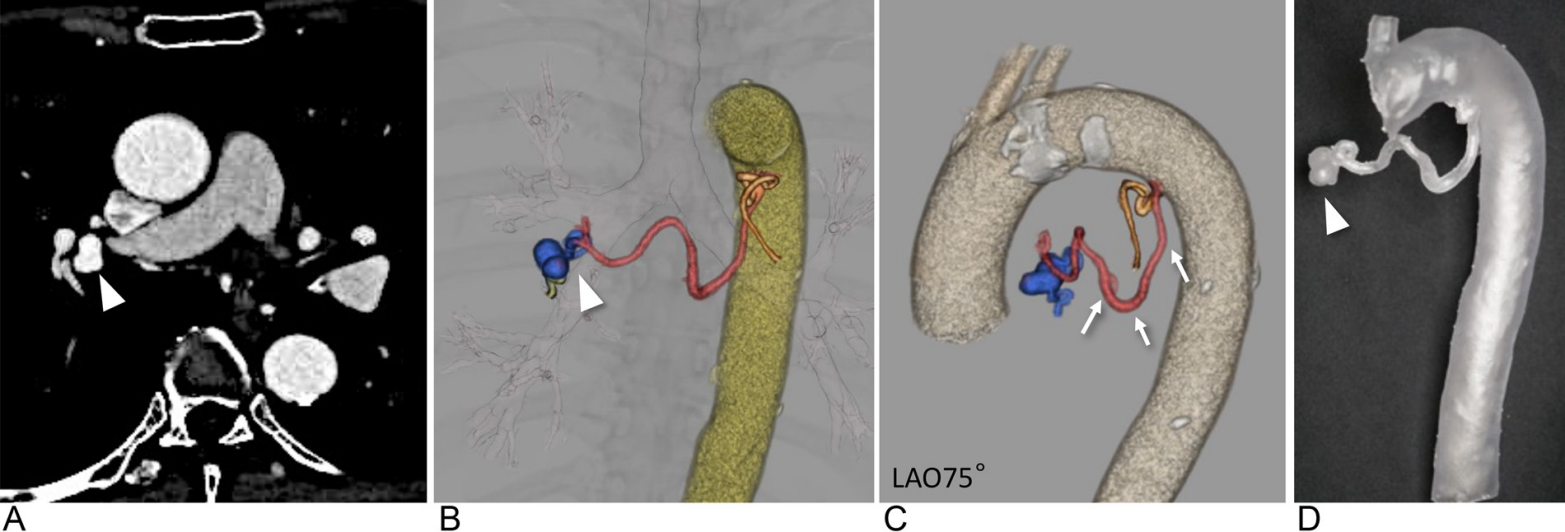
A 70-year-old female with a right bronchial artery aneurysm (Case 2). A right bronchial artery aneurysm (white arrowhead) is formed at the end of the right bronchial artery, which branches steeply from the inner curvature of the aortic arch and follows a tortuous and long course. (A) Computed tomography (CT) showing a 12 × 11 mm bronchial artery aneurysm (white arrowhead) located at the right hilum. (B and C) Volume rendering of CT angiography showing the right bronchial artery aneurysm (white arrowhead) and a white arrow showing the right bronchial artery. (D) Transparent and flexible patient-specific hollow vascular models are used for the right bronchial artery aneurysm (white arrowhead).

A 70-year-old female patient presented with a 12 × 11 mm bronchial artery aneurysm located in the right hilum (Fig. 4A and B). The patient had a history of nontuberculous mycobacteriosis. The aneurysm was incidentally detected on CT, and the patient was referred to our department for prevention of aneurysm rupture. The common trunk of the bronchial arteries arose from a sharply curved section of the descending aorta (Fig. 4C), making catheter navigation particularly difficult. The right bronchial artery was tortuous and extended over a long segment, further complicating the approach to the aneurysm for coil embolization. This made it challenging to navigate the catheter, including the microcatheter, to the aneurysm.

**Fig. 5:**
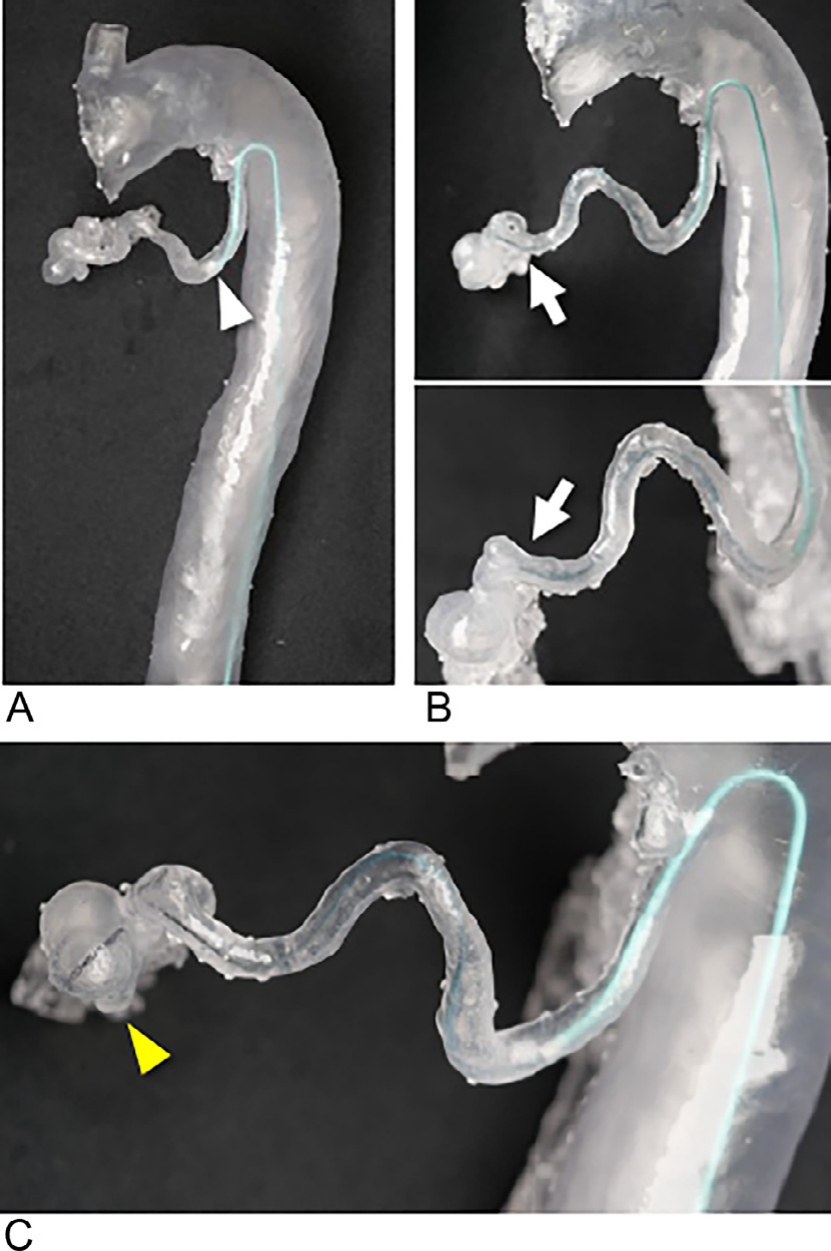
Preoperative simulation with vascular model (Case 2). (A) The 4-Fr modified Simmons catheter (white arrowhead) advanced to the right bronchial artery. (B) The 1.9-Fr microcatheter catheter advances via the 4-Fr modified Simmons catheter; the white arrow shows the tip of the microcatheter. (C) A 0.016-inch wire (yellow arrowhead) is inserted into the aneurysm via a microcatheter.

A patient-specific vascular model was created to simulate this procedure (Fig. 4D), which revealed that conventional guiding catheters, such as the Judkins left (JL), were insufficient to provide the necessary backup support for the microcatheter to reach the aneurysm (not shown). By testing various options, a 4-Fr modified Simmons catheter (80 cm) was found to be optimal, allowing stable distal navigation and successful insertion of a 1.9-Fr microcatheter (Progreat λ19, 130 cm, Terumo Clinical Supply, Tokyo, Japan) into the aneurysm within the model (Fig. 5).

**Fig. 6:**
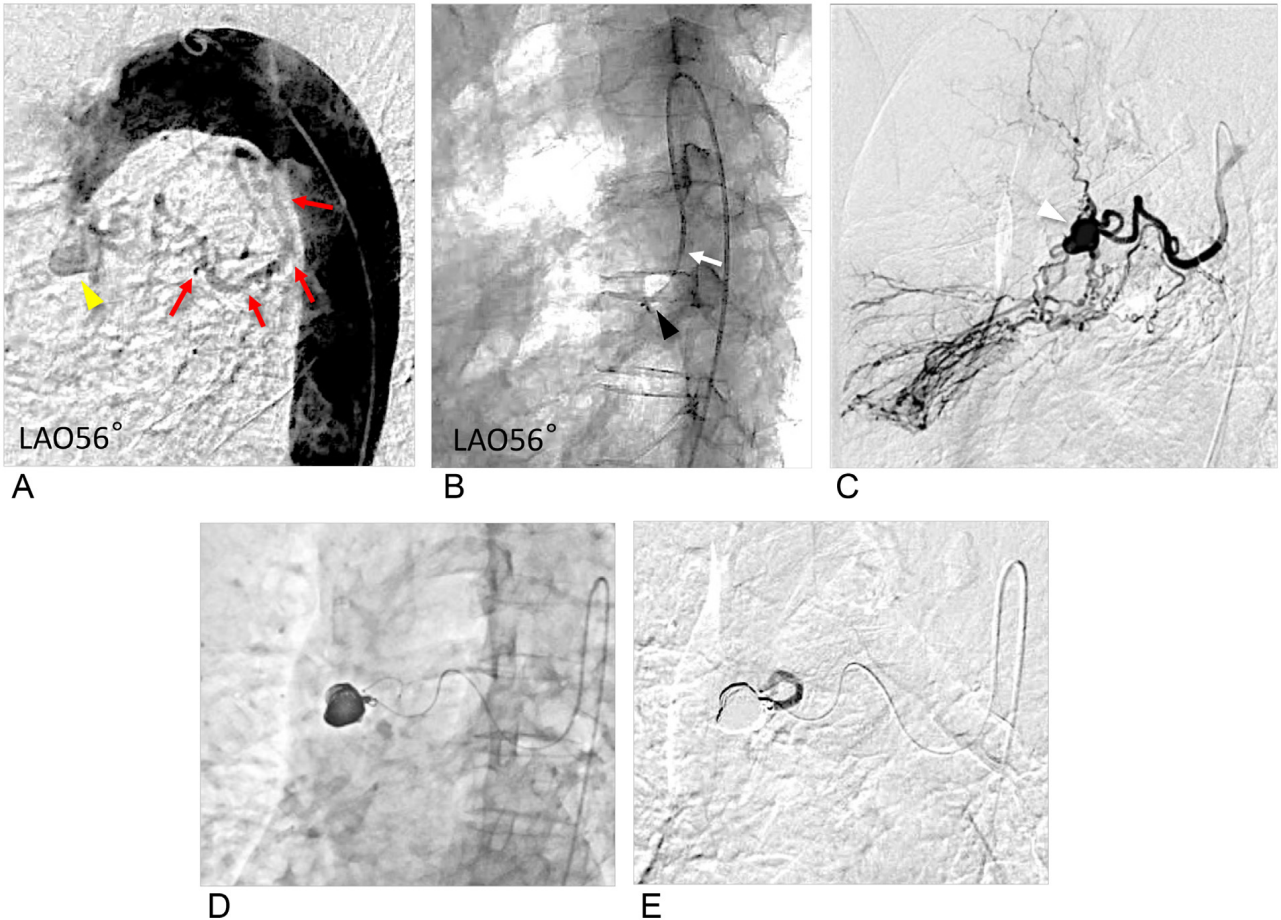
Angiography during the treatment (Case 2). (A) Pretreatment digital subtraction aortography (DSA) demonstrating a bent and tortuous right bronchial artery branching from the inner side of the aortic arch (red arrow). The yellow arrow shows the right bronchial artery aneurysm. (B) Digital angiography demonstrating that the 4-Fr modified Simmons catheter was inserted deeply into the right bronchial artery. The white arrow indicates the catheter tip, and the black arrowhead indicates the microcatheter tip. (C) DSA showing the aneurysm (white arrowhead), dilation of the bronchial artery, and vascular proliferation associated with atypical mycobacterial pneumonia and shunt with the pulmonary artery. (D) The aneurysm is packed using multiple coils. (E) After embolization, contrast media showed that the peripheral blood flow had been interrupted, indicating successful embolization.

In the patient's actual procedure, the coils for aneurysm embolization were 4 detachable coils (Target XL Detachable Coils, 9 mm × 30 cm × 2, 8 mm × 30 cm × 2) and 8 pushable coils via a microcatheter with good backup from a 4-Fr modified Simmons catheter. Embolization was successfully completed with no complications (Fig. 6).

## Discussion

Our cases demonstrated that using a 3D-printed vascular model facilitated smoother procedures by reducing catheter exchange frequency and shortening the time required for catheter engagement and placement, which are critical for minimizing patient risk in embolization procedures.

A realistic vascular model for catheter and wire manipulation requires (a) friction and flexibility similar to that of the human artery, (b) reproduction of an extensive access route including the femoral, radial, or brachial arteries, and (c) transparency that allows simulation without fluoroscopy [[9], [10], [11]]. To meet these requirements, we developed a 3D-printed vascular hollow model using a transparent, flexible resin material coated with silicone to mimic the slipperiness of biological arteries [9]. The silicone-coated, flexible resin material allowed more accurate catheter placement and movement, which is critical for simulating effective embolization. This improvement addressed the inadequate slippage often observed in conventional silicon models [12], enhancing the accuracy of catheter manipulation and enabling realistic simulation.

This patient-specific vascular model further advances preoperative simulation by closely approximating actual procedures. Its key advantage lies in its ability to reproduce a wide range of access routes using flexible, nonsolid materials with silicone coating, addressing the limitations of previous nonhollow and small-scale hollow models for peripheral aneurysms in the trunk region [[4], [5], [6], [7]]. By enabling realistic catheterization of the primary branches of the aorta, this model provides a more practical and accurate alternative to existing simulation tools. This innovation represents a key advancement over prior nonhollow and small-scale hollow models [[4], [5], [6], [7]], expanding the practical applications of preoperative simulation in complex vascular procedures.

Preoperative simulation facilitated smoother procedures by enabling optimal catheter selection, reducing the frequency of catheter exchanges, and shortening the time required for catheter engagement and placement. This approach has been shown to improve procedural efficiency and safety in various endovascular treatments [10,13,14]. Preoperative simulation for abdominal aortic aneurysm stent placement reduced procedure time, fluoroscopy time, and contrast medium usage compared with control groups [13].

The use of preoperative simulation in these cases proved effective in the preselection of the optimal catheter. In coil embolization of peripheral aneurysms of the body trunk, appropriate catheter selection with adequate backup is necessary to prevent coil migration and inadequate embolization [15,16]. This approach not only facilitated smoother procedures but also ensured adequate backup support for safe and reliable embolization.

Owing to time and cost constraints, creating individual vascular models for every preoperative simulation may not be feasible. Simulations using embolic materials are expensive; however, the catheters are relatively affordable, indicating favorable cost-effectiveness. Furthermore, reusable vascular models can be valuable for resident and student training and offer potential as a training tool for experts in reproducing complex cases.

This vascular model closely replicates biological vessels in terms of slippage but is still somewhat stiffer than actual vessels and lacks surrounding supportive tissue and blood flow [9]. Additionally, this model does not reproduce vascular responses to stimuli such as vasospasm, dilation, and elongation. Despite these limitations, this model provides sufficient quality for realistic simulation. Continued advancements in 3D printing technology may allow for models that more closely mimic actual blood vessels by incorporating softer materials and surrounding anatomical structures.

Our cases provide evidence of the feasibility and benefits of preoperative simulation using patient-specific vascular models for complex peripheral aneurysms. The realistic simulation enabled by this approach sets a foundation for its adoption in similar procedures, offering a reproducible method for improving catheterization accuracy.

## Patient consent

Informed Consent: Written informed consent was obtained from the patients for publication of this case report and any accompanying images. Consent for publication: Consent for publication was obtained for every person's data included in the study.

## Statement of Human and Animal Rights

This article does not involve animal subjects.
